# Temperature-Wise Calibration Increases the Accuracy of DNA Methylation Levels Determined by High-Resolution Melting (HRM)

**DOI:** 10.3390/ijms25105082

**Published:** 2024-05-07

**Authors:** Katja Zappe, Margit Cichna-Markl

**Affiliations:** Department of Analytical Chemistry, Faculty of Chemistry, University of Vienna, 1090 Vienna, Austria; katja.zappe@univie.ac.at

**Keywords:** high-resolution melting (HRM), pyrosequencing, DNA methylation, heterogeneous methylation, temperature-wise calibration, *MGMT*, promoter, enhancer

## Abstract

High-resolution melting (HRM) is a cost-efficient tool for targeted DNA methylation analysis. HRM yields the average methylation status across all CpGs in PCR products. Moreover, it provides information on the methylation pattern, e.g., the occurrence of monoallelic methylation. HRM assays have to be calibrated by analyzing DNA methylation standards of known methylation status and mixtures thereof. In general, DNA methylation levels determined by the classical calibration approach, including the whole temperature range in between normalization intervals, are in good agreement with the mean of the DNA methylation status of individual CpGs determined by pyrosequencing (PSQ), the gold standard of targeted DNA methylation analysis. However, the classical calibration approach leads to highly inaccurate results for samples with heterogeneous DNA methylation since they result in more complex melt curves, differing in their shape compared to those of DNA standards and mixtures thereof. Here, we present a novel calibration approach, i.e., temperature-wise calibration. By temperature-wise calibration, methylation profiles over temperature are obtained, which help in finding the optimal calibration range and thus increase the accuracy of HRM data, particularly for heterogeneous DNA methylation. For explaining the principle and demonstrating the potential of the novel calibration approach, we selected the promoter and two enhancers of *MGMT*, a gene encoding the repair protein MGMT.

## 1. Introduction

DNA methylation is an important epigenetic modification. In vertebrates, DNA methylation predominantly occurs at carbon-5 in cytosine residues that are followed by guanine in the DNA sequence, so-called CpGs. The methyl group is transferred from S-adenyl methionine (SAM), catalyzed by DNA methyltransferases (DNMTs). DNA methylation of CpGs in regulatory elements, including promoters and enhancers, plays a crucial role in gene regulation [1,2]. In addition, DNA methylation is essential for silencing retroviral elements, genomic imprinting, and X chromosome inactivation [3].

Alterations in DNA methylation have been associated with a variety of pathological conditions, including cancer [4,5]. Tumor cells frequently show global DNA hypomethylation, causing chromosomal instability, whereas the promoter region of tumor suppressor genes is often hypermethylated [6]. Cancer-specific alterations in DNA methylation have also been reported for enhancers [7,8,9,10].

Various analytical methods are applicable for DNA methylation analysis. Methods involving polymerase chain reaction (PCR) require treatment of DNA with bisulfite. Without bisulfite treatment, information on the DNA methylation status would get lost because the DNA polymerase does not distinguish between methylated and unmethylated cytosines [11]. By bisulfite treatment, unmethylated cytosines are converted to uracils and subsequently replaced by thymines during PCR, whereas methylated cytosines are protected from conversion and thus remain cytosines [12].

Methylation microarrays are the method of choice for genome-wide DNA methylation analysis at single CpG resolution [13]. The last generation of commercial methylation arrays covers over 935,000 CpG sites of the human methylome [14]. For targeting a lower number of individual CpGs in specific regions of interest, pyrosequencing (PSQ) is frequently applied. PSQ is a sequencing by synthesis method. Incorporation of the correct nucleotide results in the formation of pyrophosphate, and by exploiting several enzyme reactions, a luminescence signal can be measured in real-time [15]. PSQ is considered the gold standard for targeted DNA methylation analysis of individual CpGs. However, PSQ does not provide information on specific methylation patterns, e.g., monoallelic methylation, because the methylation status obtained for an individual CpG is the average methylation status across all alleles in the sample [16].

An alternative targeted approach is high-resolution melting (HRM) [17]. In contrast to PSQ, HRM only yields the average methylation status across all CpGs in the PCR product. However, it provides information on the methylation pattern, including the occurrence of mosaic [18] or monoallelic methylation [19]. In addition, it is highly sensitive and labor- and cost-efficient [20]. A prerequisite for HRM is that the target region is amplified in the presence of a saturating DNA intercalating dye, e.g., EvaGreen. After the last PCR cycle, the PCR products are slowly melted by changing the temperature in small increments. The intercalating dye is released and the fluorescence intensity decreases [21]. The melting behavior of the PCR products depends on various parameters, including their length and the content of guanine and cytosine. As a consequence of bisulfite treatment, PCR products originating from unmethylated DNA templates are rich in thymines and therefore melt at lower temperature than those originating from methylated DNA templates, where cytosines from CpGs are preserved [17].

In order to be able to determine the (average) methylation status of the CpGs in the target region in unknown samples, a calibration function has to be established by analyzing DNA standards of known methylation status and mixtures thereof. Most commonly, raw fluorescence data are normalized, and melt curve data are interpolated in between normalization temperature intervals [22]. Some papers report good correlation between PSQ and HRM methylation data [23,24,25]. However, others state that HRM should only be used for qualitative purposes [26], particularly in case of heterogeneous methylation [19,27,28].

Here, we present a novel calibration approach for HRM, i.e., temperature-wise calibration. With this calibration approach, the temperature interval for normalization can be set in an optimal manner. Thus, the average methylation status of CpGs in the amplicon can be obtained more accurately, particularly in case of heterogenous methylation. We selected the promoter and two enhancers of *MGMT*, a gene coding for the repair protein MGMT, to demonstrate the potential of the novel calibration approach. *MGMT* promoter methylation was selected due to its significance as a predictive biomarker for treatment response to alkylating agents such as temozolomide [29,30]. Recent findings from our research indicate that the methylation of *MGMT* enhancer regions plays a role in regulating MGMT expression and is associated with various clinical parameters in patients with glioblastoma (GBM) [9,10].

## 2. Results and Discussion

### 2.1. Principle of the Classical and the Novel HRM Calibration Approach

**Figure 1:**
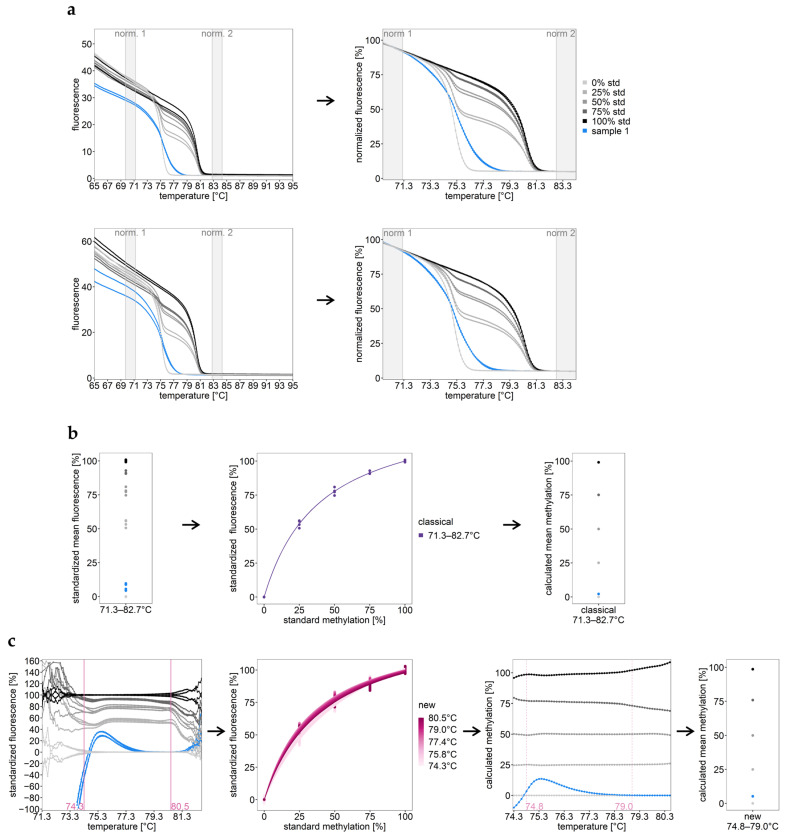
Principle of HRM data evaluation: (**a**) data normalization; (**b**) classical calibration approach; (**c**) novel calibration approach. (**a**) Raw HRM data from two independent runs are normalized over a 1.5 °C interval (gray shaded) before and after the melting region, resulting in normalized HRM plots. norm.: normalization intervals; std: standard. (**b**) In the classical approach, the whole temperature range in between the normalization intervals is used for calibration. The classical calibration approach provides the mean methylation level for each sample. (**c**) In the new approach, calibration is performed for each temperature point separately, resulting in methylation profiles over temperature. Methylation profiles over temperature allow for finding the optimal temperature range for calibration. The temperature interval between the two pink dashed lines turned out to be optimal. The novel calibration approach yields DNA methylation levels at specific temperature points. However, as for the classical calibration approach, mean methylation can be calculated as well.

The workflow of the classical and novel HRM calibration approach is shown in Figure 1. For both calibration approaches, melt curve data are used in between normalization intervals (Figure 1a) and subsequently standardized.

The classical calibration process (Figure 1b) includes the whole temperature range in between the normalization intervals. It results in one mean standardized fluorescence (SF) value across the normalized range for each PCR well. The calibration function is established by manual curve fitting using the SF values obtained for the standard series (0%, 25%, 50%, 75%, 100%). The mean DNA methylation level of the target region in a sample is calculated from this single calibration function using Equation (1).

(1) $${SF}_{sample}[\%]=\frac{{NF}_{sample}-{\bar{NF}}_{UM}\;}{{\bar{NF}}_{M}-{\bar{NF}}_{UM}}*100$$

SF_sample_: standardized fluorescence of the sample;

NF_sample_: normalized fluorescence of the sample;

${\bar{NF}}_{UM}$: mean normalized fluorescence of the unmethylated standard;

${\bar{NF}}_{M}$: mean normalized fluorescence of the methylated standard.

The standards used for calibration consist of fully methylated and/or unmethylated strands, resulting in two distinct melting transitions (melt peaks in negative derivatives) of the melt curves. Melt curves obtained for heterogeneously methylated strands differ in shape compared to those of homogeneously methylated DNA standards. In case of co-occurrence of unmethylated strands and strands with low heterogeneous methylation, melt curves are even more complex, increasing the inaccuracy of calculated DNA methylation levels.

The novel calibration approach (Figure 1c) is based on performing calibration for each individual temperature point. The normalized fluorescence (NF) for each PCR well is standardized for each individual temperature point t (temp t) using the mean NF from both PCR wells of the unmethylated (UM) and methylated (M) standard, respectively (Equation (2)).

(2) $${SF}_{sample\;at\;temp\;t}[\%]=\frac{{NF}_{sample\;at\;temp\;t}-{\bar{NF}}_{UM\;at\;temp\;t}\;}{{\bar{NF}}_{M\;at\;temp\;t}-{\bar{NF}}_{UM\;at\;temp\;t}}*100$$

The novel calibration approach has several advantages compared to the classical one. By using a for-loop for solving multiple calibration functions (for each temperature point one), methylation profiles over temperature can be calculated. These methylation profiles over temperature help in finding the optimal temperature range for calibration. In addition, methylation profiles over temperature provide a more detailed picture of the methylation of the target region. Methylation levels at specific temperature points may be used for statistical analyses, e.g., for identifying potential biomarkers. Mean DNA methylation across the calibrated temperature range, as obtained via the classical approach, can be calculated as well. In this study, we calculated the mean DNA methylation status in order to compare results from different calibration approaches and/or results from HRM and PSQ. In the latter case, the mean of PSQ data obtained for individual CpGs had to be calculated as well.

### 2.2. Potential of the Novel Calibration Approach

The potential of the novel calibration approach will be demonstrated for three assays, targeting the promoter and two enhancers of *MGMT*, respectively. These assays were selected because their target regions differ in length, GC content, and/or number of CpGs (Table S1). In both enhancers, the GC content and the number of CpGs is lower than in the promoter. In addition, the assays vary in terms of the difference in the melting temperature between fully methylated and unmethylated DNA strands. Furthermore, the assays for the *MGMT* promoter and enhancer 3 were performed with a commercial kit; the master mix for enhancer 2 was prepared my mixing commercially available reagents in-house.

The potential of the novel calibration approach will be demonstrated on two sample sets, consisting of 20 commercial cell lines (sample set 1) and 41 human tumor cell lines established from glioma patients (sample set 2).

#### 2.2.1. MGMT Promoter Assay

The target region of the *MGMT* promoter assay is 98 bp long, has a GC content of 47%, and contains 12 CpGs.

**Figure 2:**
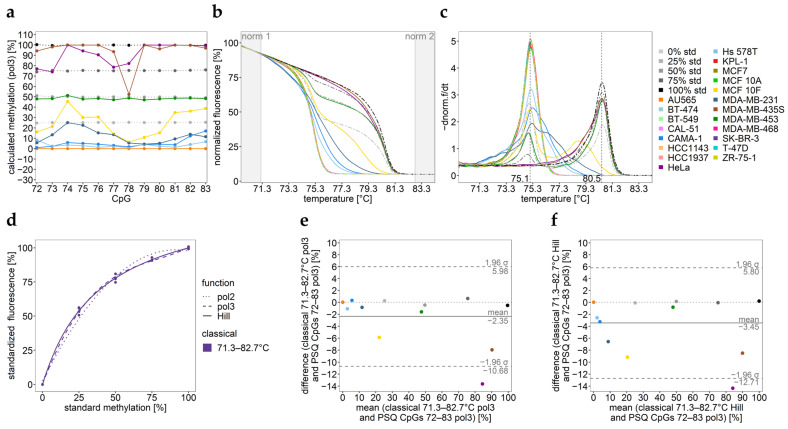
DNA methylation analysis of the *MGMT* promoter in commercial cell lines. (**a**) DNA methylation levels of individual CpGs 72–83 determined by PSQ. (**b**) Normalized melt curves and (**c**) their negative derivative obtained by HRM analysis. norm.: normalization intervals; std.: standard. Black dashed lines show the melting temperatures (Tm) for the unmethylated and fully methylated strand, respectively. Result for one representative well of two independent runs is shown. (**d**) Calibration curves obtained by the classical approach are shown (three regression functions: pol 2, pol 3, Hill; the whole temperature range (71.3–82.7 °C) was used). (**e**,**f**) Bland–Altman plots, indicating the agreement between HRM data using the classical calibration approach (71.3–82.7 °C) by applying (**e**) pol 3 and (**f**) Hill function and the mean DNA methylation of individual CpGs determined by PSQ (pol 3).

Analysis of the *MGMT* promoter for sample set 1—consisting of 20 commercial cell lines, by PSQ and HRM resulted in DNA methylation levels of individual CpGs (Figure 2a) and normalized HRM curves (Figure 2b) and their negative derivative (Figure 2c), respectively. The difference between the melt peaks obtained for methylated and unmethylated DNA strands was 5.4 °C. Both PSQ and HRM data indicate that the target region was unmethylated in almost all samples.

Only in seven cell lines (CAMA-1, HeLa, Hs 578T, MCF 10F, MDA-MB-231, MDA-MB-435S, and MDA-MB-453) does the target region show methylation levels >3% (PSQ). Both PSQ and HRM indicate that the target region is methylated rather heterogeneously, except for MDA-MB-453 (Figure 2a–c). By applying the classical approach, the assay was calibrated using the whole normalized temperature range (71.3–82.7 °C) (Figure 2d). For higher heterogeneously methylated samples, HeLa and MDA-MB-435S, the mean methylation levels obtained by the classical approach were in less agreement with the mean DNA methylation of individual CpGs determined by PSQ than homogeneously methylated samples, as indicated by the Bland–Altman plots (Figure 2e,f) and Table 1. In these cases, the inaccuracy of data is mainly caused by the broad and complex shape of the melt curves, resulting from heteroduplexes due to base-pairing mismatches between heterogeneously methylated strands. These heteroduplexes melt at lower temperatures than the unmethylated DNA standard, resulting in the underestimation of the methylation status.

**Figure 3:**
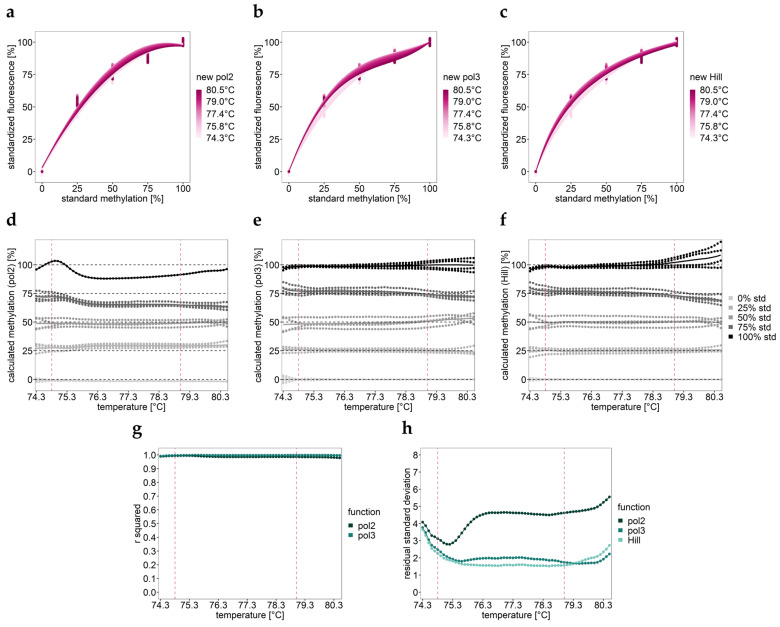
Suitability of different function types for HRM by applying the new calibration approach. Assay for the *MGMT* promoter. (**a**–**c**) Calibration curves obtained by temperature-wise calibration by (**a**) pol 2, (**b**) pol 3, and (**c**) Hill function. (**d**–**f**) Methylation profiles over temperature obtained for standards (std) using (**d**) pol 2, (**e**) pol 3, and (**f**) Hill function. The temperature interval between the two pink dashed lines turned out to be most suitable for calibration. Solid horizontal line: mean; dashed line: expected methylation level for the respective standard. (**g**,**h**) Evaluation of the curve fits by (**g**) r squared (not applicable for non-linear functions) and (**h**) residual standard deviation.

Calibration curves obtained by the novel approach are shown in Figure 3. By testing various regression functions, including loess, linear, polynomial grade 2 (pol 2, Figure 3a), polynomial grade 3 (pol 3, Figure 3b), and Hill (Figure 3c), pol 2, pol 3, and Hill appeared to be suitable for calibration. Pol 3 and Hill were found to be more suitable because they resulted in low residual standard deviations (Figure 3h). Pol 2 led to high residual standard deviations, particularly at higher temperatures (Figure 3h).

**Figure 4:**
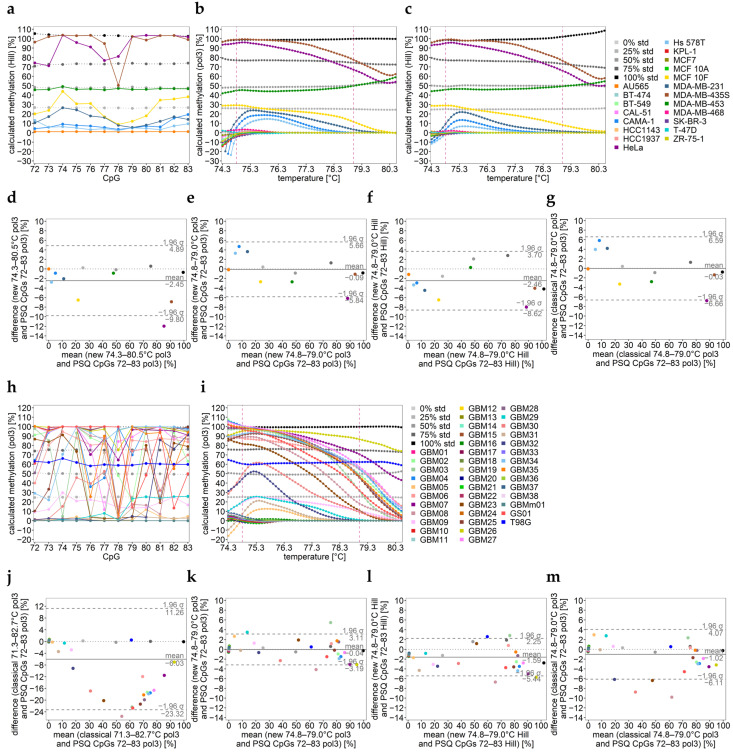
DNA methylation analysis of (**a**–**g**) sample set 1 (commercial cell lines) and (**h**–**m**) sample set 2 (glioma cell lines) for the *MGMT* promoter. (**a**,**h**) DNA methylation levels of individual CpGs 72–83 determined by PSQ. (**b**,**c**,**i**) Methylation profiles over temperature obtained by temperature-wise calibration. The temperature interval between the two pink dashed lines turned out to be most suitable for calibration. Solid horizontal line: sample; dotted line: standard. (**d**–**g**,**j**–**m**) Bland–Altman plots indicating the agreement between data. Data points represent the mean of two independent PCR runs.

The respective methylation profiles over temperature (Figure 3d–f) obtained by temperature-wise calibration were used to find the optimal calibration range, aiming at increasing the accuracy of results for heterogeneously methylated samples. Our strategy was to exclude melt curve regions containing heteroduplexes (lower temperatures) and those where curves from the standards were not distinguishable from each other (mainly higher temperatures) (Figure 1c and Figure 3d–f). By testing the applicability of several temperature ranges differing in their width, the range 74.3–80.5 °C (Figure 3d–f) was found to be suitable, indicated by the good agreement with PSQ data (Figure 4d). The best agreement was found for an even narrower temperature range (74.8–79.0 °C, Figure 4e,f). Notably, by applying the optimal temperature range, the classical calibration approach also yielded good agreement with mean methylation values obtained by PSQ (Figure 4g, Table 1).

Methylation profiles over temperature cannot only be used to find the optimal temperature range for calibration. They also yield information on the methylation pattern of the target sequence (Figure 3d–f and Figure 4b,c). For MDA-MB-453 (dark green), the methylation profile over temperature indicates monoallelic methylation. The occurrence of monoallelic methylation cannot be verified by PSQ because PSQ does not yield information on the methylation pattern of individual strands. However, for all individual CpGs, a methylation level of 50% was obtained, underlying the occurrence of monoallelic methylation in MDA-MB-453. The methylation profile over temperature for MCF 10F (yellow) suggests the co-occurrence of unmethylated and heterogeneously methylated strands since, in the low temperature range, the methylation profile is parallel to the 25% methylated standard due to the specific melting transition of the unmethylated allele (Figure 2b,c and Figure 4b,c).

In most glioma cell lines (sample set 2), the *MGMT* promoter was unmethylated (20 out of 41 samples) or methylated heterogeneously (20 out of 41 samples). Both the Bland–Altman plot (Figure 4j–l) and Table S1 indicate that by applying the novel calibration approach, the accuracy of HRM data improved drastically for 39% and remained similar for 61% of the samples. By narrowing the temperature range, the accuracy of the data obtained by the classical approach also improved (Table 2 and Table S1, Figure 4m), as observed for sample set 1. Methylation profiles over temperature hint at monoallelic methylation for T98G and the co-occurrence of unmethylated and low heterogeneous methylated strands for GBM09 and GBM29.

#### 2.2.2. MGMT Enhancer 2 Assay

The assay developed for *MGMT* enhancer 2 targets a 137 bp long sequence, having a GC content of 33% and containing eight CpGs.

**Figure 5:**
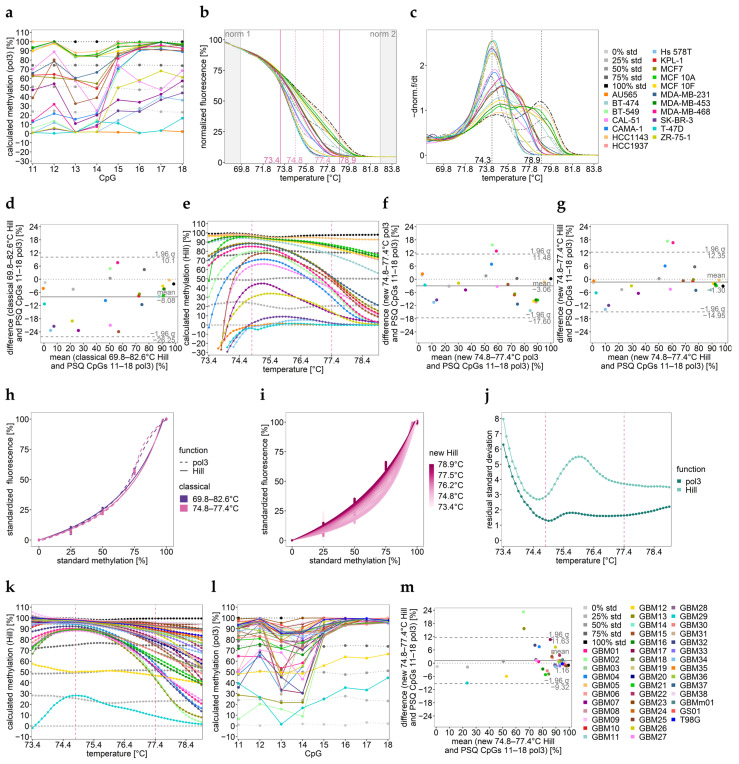
DNA methylation analysis of (**a**–**g**) sample set 1 (commercial cell lines) and (**h**–**m**) sample set 2 (glioma cell lines) for *MGMT* enhancer 2. (**a**,**l**) DNA methylation levels of individual CpGs 11–18 determined by PSQ. (**b**) Normalized melt curves and (**c**) their negative derivative obtained for commercial cell lines obtained by HRM analysis. norm.: normalization intervals. The black dashed lines shows the melting temperatures (Tm) for the unmethylated and fully methylated strand. One representative well for two independent runs is shown. (**e**,**k**) Methylation profiles over temperature obtained by new-temperature-wise calibration for HRM data. The temperature interval between the two pink dashed lines turned out to be most suitable for calibration. Solid horizontal line: sample; dotted line: standard. (**h**) Calibration curves for the classical approach. (**i**) Calibration curve for the novel approach. (**j**) Evaluation of the curve fits by residual standard deviation. (**d**,**f**,**g**,**m**) Bland–Altman plots indicating the agreement between data. Data points represent the mean of two independent PCR runs.

PSQ of commercial cell lines (sample set 1) indicated that the eight CpGs were methylated rather heterogeneously (Figure 5a). Differences in the shape of the normalized HRM curves (Figure 5b) and their negative derivative (Figure 5c) also hint at heterogeneous methylation. The melt temperature of fully methylated DNA strands was 4.6 °C higher than that of unmethylated DNA strands.

By applying the classical calibration approach, the methylation status was drastically underestimated in 65% of the samples (Figure 5d, Table S2).

The methylation profiles over temperature obtained by the novel calibration approach suggest an optimal calibration range of 74.8–77.4 °C, resulting in good agreement with PSQ for 80% of the samples (Table S2). By narrowing the calibration range, similar results were also obtained with the classical approach (Table 3).

However, for two particularly heterogeneously methylated cell lines (PSQ: mean~50%, SD > 40%), BT-549 and MDA-MB-468, mean DNA methylation levels determined by the novel calibration approach were too high, and for Hs 578T and SK-BR-3 (PSQ: mean~18%, SD~16%), they were still too low compared to the mean methylation determined by PSQ. For methylated samples, methylation profiles over temperature were not horizontal (Figure 5e), hinting at heterogeneous methylation.

Calibration curves obtained by both calibration approaches are shown in Figure 5h,i. Hill was found to be more suitable for calibration than pol 3, especially for higher methylation levels (Figure 5h–j).

In glioma samples (sample set 2), the *MGMT* enhancer 2 region was also heterogeneously methylated, except for GBM12 (yellow, Figure 5k,l). The methylation profile of GBM12 hints at the co-occurrence of an unmethylated and a heterogeneously methylated strand. According to PSQ data, CpGs 15–18 showed a higher methylation status than CpGs 11–14 (Figure 5l).

The Bland–Altman plot (Figure 5m), Table 4 and Table S3 indicate that by calibrating in the optimal temperature interval (74.8–77.4 °C), the novel and the classical calibration approach afforded methylation data that were in good agreement with the mean methylation data obtained by PSQ (93% of the samples). For the three samples being very heterogeneously methylated, GBM02 and GBM13 (PSQ: mean~55%, SD > 36%) and GBM17 (PSQ: mean = 80%, SD = 30%), the methylation status was overestimated. Since glioma samples were more highly methylated, HRM data were in better agreement with the mean PSQ data than those obtained for sample set 1.

#### 2.2.3. MGMT Enhancer 3 Assay

The target region of *MGMT* enhancer 3 is 138 bp long, has a GC content of 38%, and contains eight CpGs.

**Figure 6:**
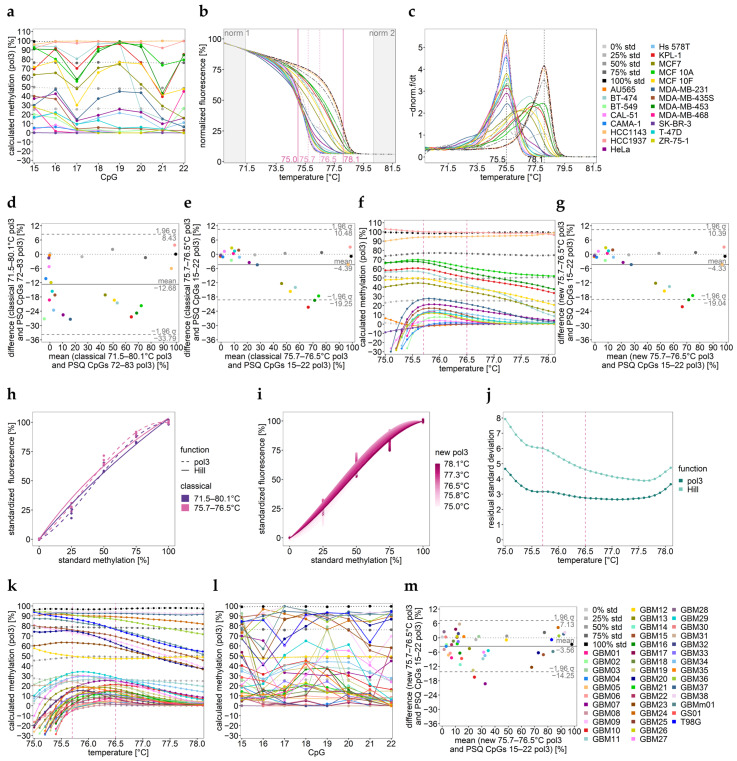
DNA methylation analysis of (**a**–**g**) sample set 1 (commercial cell lines) and (**h**–**m**) sample set 2 (glioma cell lines) for *MGMT* enhancer 3. (**a**,**l**) DNA methylation levels of individual CpGs 15–22 determined by PSQ. (**b**) Normalized melt curves and (**c**) their negative derivative obtained for commercial cell lines obtained by HRM analysis. norm.: normalization intervals. Black dashed lines show the melting temperatures (Tm) for the unmethylated and fully methylated strands. One representative well for two independent runs is shown. (**f**,**k**) Methylation profiles over temperature obtained by new temperature-wise calibration for HRM data. The temperature interval between the two pink dashed lines turned out to be most suitable for calibration. Solid horizontal line: sample; dotted line: standard. (**h**) Calibration curves for the classical approach. (**i**) Calibration curve for the novel approach. (**j**) Evaluation of the curve fits by residual standard deviation. (**d**,**e**,**g**,**m**) Bland–Altman plots indicating the agreement between data. Data points represent the mean of two independent PCR runs.

Both PSQ and HRM data indicate that in commercial cell lines (sample set 1), the target region was methylated rather heterogeneously (Figure 6a–c). Fully methylated DNA strands melted at only 2.6 °C higher than unmethylated DNA strands. Moreover, both the normalized melt curves and their negative derivatives hint at an additional melt domain at 71.5 °C.

Thus, mean methylation levels determined by the classical calibration approach differed from the mean calculated from the PSQ data determined for individual CpGs (Table 5). Deviations from mean PSQ values > 20% were observed for the low-, moderate-, and high-methylation range in 40% of the samples (Figure 6d).

However, when the temperature range used for calibration was severely narrowed to 75.7–76.5 °C, not only the novel calibration approach but also the classical one increased the accuracy for 85% of the samples (Figure 6e–g; Table 5 and Table S4). For none of the samples, the accuracy decreased. Only for one rather heterogeneously methylated sample, KPL-1 (red), was a deviation from mean PSQ values > 20% still observed (PSQ: mean = 78%; SD = 19% (Figure 6a,e–g)). For calibration, both pol3 and Hill turned out to be suitable; however, pol 3 led to slightly better results (Figure 6h–j).

In general, calibration by including the whole temperature range in between the normalization intervals led to too-low DNA methylation values in 80% of the samples in sample set 2. Narrowing the calibration range improved the accuracy of HRM data obtained for theses samples (Figure 6k–m, Table 6 and Table S5). For none of the samples, a deviation from mean PSQ values > 20% was observed using the narrowed range.

By applying the optimal, very narrow range (75.7–76.5 °C), mean HRM data of 88% of the samples were in good agreement with mean PSQ data.

Methylation profile over temperature for GBM12 (yellow) overlapped with that of the 50% standard, indicating monoallelic methylation (Figure 6k).

#### 2.2.4. Guidelines for Selecting the Optimal Calibration Range

The data presented above indicate clearly that the temperature range used for calibration has a strong impact on the accuracy of DNA methylation levels determined by HRM. Our data indicate that calibration should start at the temperature point, at which the profile for low heterogeneously methylated samples crosses that of the unmethylated standard, or at least, does not deviate too drastically to the negative. In case low heterogeneously methylated samples are not available, we suggest starting calibration slightly below the melting temperature of the unmethylated standard. For the end of the calibration range, we suggest the following rule of thumb: the end of the calibration range should be set at Tm of the methylated strands minus a °C, with a being 1.5 × ∆ Tm if the difference in the melt temperature between fully methylated and unmethylated strands is >2 °C, and 0.5 × ∆Tm if the difference is ≤2 °C.

#### 2.2.5. Statistical Analyses by Using Methylation Data for Specific Temperature Points

**Figure 7:**
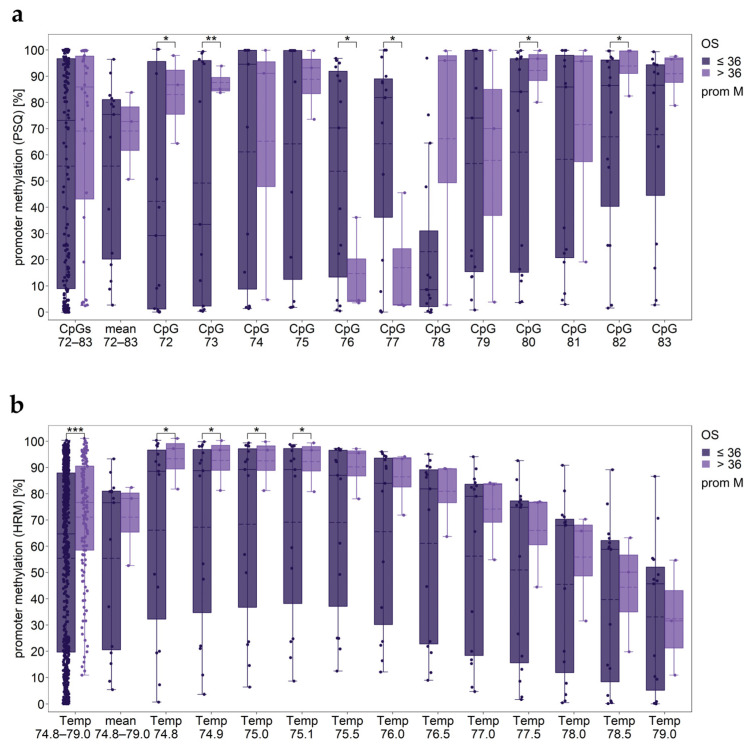
Potential of using methylation data for each temperature point for statistical analyses. *MGMT* promoter methylation levels for non-long-term survivors (≤36 months) and long-term survivors (>36 months). (**a**) PSQ; (**b**) HRM. prom M: promoter methylated. Significance levels: * *p* ≤ 0.05, ** *p* ≤ 0.01, *** *p* ≤ 0.001.

The novel calibration approach yields methylation data for each temperature point. These data can be used for statistical analyses, e.g., with clinical parameters, as shown for a dataset obtained for the *MGMT* promoter by analyzing stable GBM cell lines [10]. PSQ indicated that long-term survivors (>36 months) showed significantly higher methylation of CpGs 72, 73, 80, and 82, and significantly lower methylation of CpGs 76 and 77 compared to non-long-term survivors (≤36 months) (Figure 7a). The mean methylation level determined by HRM (novel calibration approach using the optimal temperature range) was not significantly different between the two groups. However, for four temperature points, a significant difference between long-term survivors and non-long-term survivors was found.

## 3. Materials and Methods

### 3.1. Standards and DNA Extracts

Human unmethylated and methylated DNA was provided by Zymo Research, USA. The study included DNA extracts from 18 commercial human cancer cell lines (AU565, BT-474, BT-549, Cal-51, CAMA-1, HCC1143, HCC1937, HeLa, Hs 578T, KPL-1, MCF7, MDA-MB-231, MDA-MB-435S, MDA-MB-453, MDA-MB-468, SK-BR-3, T-47D, and ZR-75-1) and two non-cancerous cell lines (MCF 10A, MCF 10F) [31,32]. In addition, a second samples set, including DNA extracts from primary human tumor cell lines established from 40 glioma patients [9] and the commercial glioblastoma cell line T98G, was analyzed. DNA was isolated using common commercial DNA extraction kits. DNA extracts were stored at −20 °C.

For sodium bisulfite conversion of unmethylated cytosines, the EpiTect Fast Bisulfite Conversion Kit (Qiagen, Hilden Germany) was used according to the manufacturer’s protocol. Converted DNA was quantified with the Qubit ssDNA Assay Kit (Thermo Fisher Scientific, Vienna, Austria) and stored at −20 °C until PCR.

### 3.2. Target Regions and Assays

The primer sets targeting two *MGMT* enhancers, enhancer 2 (46 CpGs, identified by Chen et al. [33]) and enhancer 3 (hs699, 33 CpGs [34]), were developed in-house [9]. For investigating the methylation status of the *MGMT* promoter, a primer set targeting CpGs 72–83 out of 98 CpGs was taken from literature [35]. An overview of the location and CpGs covered by the primer sets for enhancer 2, enhancer 3, and the promoter region in this study is given in Figure S1, and primer details are listed in Table S6.

### 3.3. PCR and HRM

Each PCR reaction was performed in a total volume of 20 µL, consisting of 1× PCR mix including EvaGreen HRM dye, forward and reverse primer, and 5 ng of bisulfite converted DNA using the Rotor-Gene Q instrument with 72-well rotor (Qiagen). PCR conditions, which we have optimized in our previous study [9], are listed in Table S7. For the *MGMT* promoter and enhancer 3, the EpiTect HRM Master Mix (Qiagen) in RNase-free water was used. The assay for enhancer 2 was performed by using a PCR master mix consisting of 2.5 U HotStarTaq DNA Polymerase (Qiagen) in 1× supplied PCR Buffer, 200 nM of each dNTP (PCR grade dNTP mix, Qiagen), and 1× EvaGreen dye (Biotium, CA, USA) in RNase-free water. For all assays, the following HRM program was applied directly after final elongation: strand separation for 1 min at 95 °C, strand hybridization for 1 min at 40 °C, and HRM with a ramp from 65 °C to 95 °C with 0.1 °C/hold (2s) and gain optimization (70% before melt).

Each PCR run included a methylation standard series containing bisulfite-converted human non-methylated and methylated DNA (Zymo Research, CA, USA), their 25%, 50%, and 75% mixtures, and a no-template control (2 µL nuclease-free H_2_O). Samples were analyzed in two wells per PCR and in two independent PCR runs.

HRM melt curves were assessed, normalized, and exported using Rotor-Gene Q Series Software 2.3.1 (Qiagen). Derivative melt curves were calculated from the normalized melt curves by applying Savitzky–Golay filtering for third-degree polynomials. R commands applied for the novel calibration approach are given in the Supplementary_R_file novel calibration approach.

### 3.4. PSQ of PCR Products

PSQ was performed using the PyroMark Q24 Vacuum Workstation and PyroMark Q24 Advanced instrument with PyroMark Q24 Advanced Accessories, Pyro-Mark Q24 Advanced CpG Reagents (all Qiagen), and Sepharose High Performance beads (GE Healthcare; Thermo Fisher Scientific) according to the manufacturer’s instructions. Dispensation orders were published previously [9]. PSQ data were evaluated and exported with the PyroMark Q24 Advanced software 3.0.0 (Qiagen). DNA methylation levels obtained by PSQ ≤ 5% (lower limit of quantification, LLOQ) and ≥95% (upper limit of quantification, ULOQ) were substituted with default values, namely, 2.5% and 97.5%, respectively, as proposed previously [36].

For better comparability of HRM and PSQ data, PSQ assays were calibrated. The main aim was to eliminate differences due to PCR bias. Calibration was conducted for each CpG separately using R based on signals for unmethylated and methylated DNA standards and their 25%, 50%, and 75% mixtures.

### 3.5. Data Analysis and Statistics

Data were analyzed and presented graphically using R version 3.6.2 [37]. R-packages used, including ggplot2, polynom, rstatix, are listed in supplementary file R packages.

Bland–Altman analysis was applied to investigate the agreement between data obtained by different calibration approaches or between mean methylation data obtained by HRM and PSQ. Differences ≤ 10% were considered as good agreement. Limits of agreement (LOA) were defined as mean ± 1.96 × standard deviation (σ).

## 4. Conclusions

We presented a novel calibration approach for DNA methylation analysis by HRM. The novel calibration approach is not considerably more time-consuming and offers several advantages compared to the classical calibration approach. We showed that by temperature-wise calibration, methylation profiles over temperature are obtained. These profiles help elucidate the optimal temperature range for calibration. Our data indicate that, in general, a narrow calibration range results in more accurate results compared to calibration including the whole temperature range in between the normalization intervals. This holds particularly true for samples with low and moderate heterogeneous methylation. Among the regression functions tested, pol3 was found to be suitable for all regions tested, but Hill is frequently a suitable alternative.

Methylation profiles over temperature yield information on the methylation pattern, e.g., the occurrence of monoallelic methylation.

Moreover, methylation profiles over temperature provide methylation data for each temperature point, allowing us to use these data for statistical analyses, e.g., with clinical parameters.

Although we demonstrated that the novel calibration approach results in DNA methylation levels that are in good agreement with values obtained by PSQ, we would like to stress that PSQ remains the gold standard for targeted DNA methylation analysis, providing the DNA methylation levelsof individual CpGs.

## Figures and Tables

**Table 1:** Mean DNA methylation levels (%) of the *MGMT* promoter for selected commercial cell lines obtained by PSQ and HRM by applying different calibration approaches. Calibration function: pol 3. For the remaining twelve unmethylated samples, mean methylation ranged from −0.9% to 0.8%.

Sample	Mean Methylation [%]			
	PSQ (CpGs 72–83)	Classical Calibration (71.3–82.7 °C)	Classical Calibration (74.8–79.0 °C)	Novel Calibration (74.8–79.0 °C)
AU565	0.0	0.0	−0.1	−0.2
Hs 578T	3.3	2.2	7.3	6.7
CAMA-1	5.4	5.7	11.3	10.1
MDA-MB-231	12.1	11.3	16.3	15.8
MCF 10F	25.0	19.1	21.7	22.3
MDA-MB-453	48.3	46.8	45.5	45.6
HeLa	91.2	77.6	84.4	85.1
MDA-MB-435S	94.2	86.2	92.8	93.1

**Table 2:** Mean DNA methylation levels (%) of the *MGMT* promoter for selected glioma cell lines obtained by PSQ and HRM by applying different calibration approaches. Calibration function: pol 3. For results of the whole sample set, see Table S1.

Sample	Mean Methylation [%]			
	PSQ (CpGs 72–83)	Classical Calibration (71.3–82.7 °C)	Classical Calibration (74.8–79.0 °C)	Novel Calibration (74.8–79.0 °C)
GBM31	8.8	5.4	8.7	8.6
GBM09	18.1	15.3	18.7	19.4
GBM23	50.7	30.5	44.3	52.6
T98G	60.8	61.4	61.4	61.3
GBM08	66.8	41.2	57.0	62.6
GBM03	72.7	50.4	75.1	78.2
GBM07	91.2	79.7	87.7	88.2

**Table 3:** DNA methylation levels (%) of *MGMT* enhancer 2 for selected commercial cell lines obtained by PSQ and via the classical, and the novel calibration approach. Calibration functions: pol 3 for PSQ and Hill for HRM. For the whole sample set, see Table S2.

Sample	Mean Methylation [%]			
	PSQ (CpGs 11–18)	Classical Calibration (69.8–82.6 °C)	Classical Calibration (74.8–77.4 °C)	Novel Calibration (74.8–77.4 °C)
AU565	1.5	−2.7	0.7	0.6
SK-BR-3	18.3	−3.2	6.3	6.3
ZR-75-1	31.0	12.0	27.3	27.9
BT-549	47.8	52.7	66.3	65.2
MDA-MB-435S	68.7	44.8	68.4	68.0
MCF 10A	94.7	87.2	92.2	91.7
HCC1143	95.6	95.2	95.5	95.2

**Table 4:** DNA methylation levels (%) of *MGMT* enhancer 2 region for selected GBM cell lines obtained by PSQ and via the classical, and the novel calibration approach. Calibration functions: pol 3 for PSQ and Hill for HRM. For results of the whole sample set, see Table S3.

Sample	Mean Methylation [%]			
	PSQ (CpGs 11–18)	Classical Calibration (69.8–82.6 °C)	Classical Calibration (74.8–77.4 °C)	Novel Calibration (74.8–77.4 °C)
GBM29	27.8	15.2	17.8	18.8
GBM12	55.8	48.8	49.7	49.8
GBM13	58.2	66.9	76.3	73.9
GBM01	76.7	72.5	79.2	77.4
GBM26	86.0	89.8	94.1	93.3
GBM18	87.0	77.0	83.0	82.1
GBM34	90.5	87.8	89.8	88.9
GBM03	96.7	96.8	97.3	97.1

**Table 5:** DNA methylation levels (%) of *MGMT* enhancer 3 region for selected commercial cell lines obtained by PSQ and via the classical, and the novel calibration approach. Calibration function: pol 3. For results of the whole sample set, see Table S4.

Sample	Mean Methylation [%]			
	PSQ (CpGs 15–22)	Classical Calibration (71.5–80.1 °C)	Classical Calibration (75.7–76.5 °C)	Novel Calibration (75.7–76.5 °C)
AU565	−0.1	−0.6	−1.1	−1.1
CAMA-1	1.9	−8.4	0.7	0.6
MDA-MB-468	9.2	−10.1	9.5	9.6
HeLa	23.1	−2.4	19.6	19.7
MCF7	52.3	35.1	39.9	40.1
BT-474	63.5	43	49.6	49.9
KPL-1	77.6	51.2	55.3	55.5
HCC1143	99.1	93.1	94.8	94.8

**Table 6:** DNA methylation levels (%) of *MGMT* enhancer 3 region for selected GBM cell lines obtained by PSQ and via the classical, and the novel calibration approach. Calibration function: pol 3. For results of the whole sample set, see Table S5.

Sample	Mean Methylation [%]			
	PSQ (CpGs 15–22)	Classical Calibration (71.5–80.1 °C)	Classical Calibration (75.7–76.5 °C)	Novel Calibration (75.7–76.5 °C)
GBM04	4.8	−1.1	1.8	1.2
GBM18	9.1	1.9	7.9	7.8
GBM31	10.3	7.1	15.4	16.0
GS01	19.0	0.7	12.5	10.4
GBM03	27.4	15.3	27.6	28.0
GBM10	32.6	9.0	18.1	16.2
GBM12	49.0	49.3	48.0	48.1
GBM09	75.9	57.5	69.3	69.6
GBMm01	86.7	67.8	80.7	81.0
GBM37	91.2	92.3	92.8	92.7

## Data Availability

The datasets generated during the current study are available from the corresponding author on reasonable request.

## Supplementary Materials

The following supporting information can be downloaded at https://www.mdpi.com/article/10.3390/ijms25105082/s1.
