# Supplementary material for: Temperature-Wise Calibration Increases the Accuracy of DNA Methylation Levels Determined by High-Resolution Melting (HRM)
Source: Int J Mol Sci. 2024 May 7;25(10):5082. doi: 10.3390/ijms25105082 (PMC11121480; doi:10.3390/ijms25105082)
Supplement: Supplementary file 1 [file ijms-25-05082-s001.zip › Supplementary_Figures.pdf]

Supplementary Figures

Temperature-Wise Calibration Increases the Accuracy of DNA Methylation Levels Determined by High-Resolution Melting (HRM)

Katja Zappe and Margit Cichna-Markl *

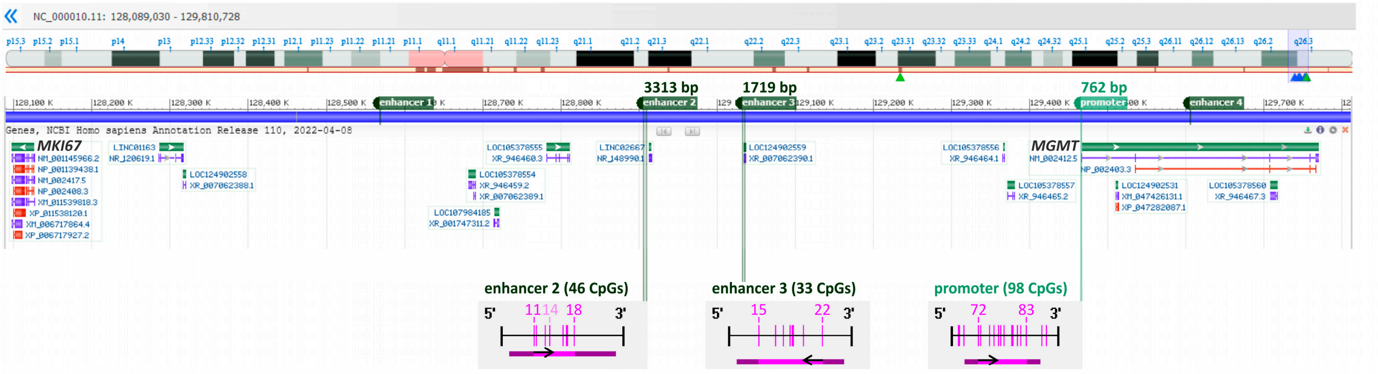

Figure S1. Overview of the location and CpGs covered by the primer sets for the *MGMT* enhancer 2, enhancer 3 and the promoter region located between the *MKI67* and *MGMT* gene on chromosome 10. Pink vertical lines indicate CpG positions, purple horizontal bars represent the region targeted by PCR-HRM, and pink horizontal bars highlight the CpGs (pink) targeted by pyrosequencing. Small black arrows symbolize the sequencing direction related to the upper strand. CpGs are numbered according to their position in the respective enhancer/promoter. Representation of chromosome 10 including gene location was taken from NCBI Genome Data Viewer and CpG line schemes were generated using the Methyl Primer Express Software v1.0 (Thermo Fisher Scientific) and adapted manually.
