# Supplementary material for: Temperature-Wise Calibration Increases the Accuracy of DNA Methylation Levels Determined by High-Resolution Melting (HRM)
Source: Int J Mol Sci. 2024 May 7;25(10):5082. doi: 10.3390/ijms25105082 (PMC11121480; doi:10.3390/ijms25105082)
Supplement: Supplementary file 1 [file ijms-25-05082-s001.zip › Supplementary_Tables.pdf]

Supplementary Tables

Temperature-Wise Calibration Increases the Accuracy of DNA Methylation Levels Determined by High-Resolution Melting (HRM)

Katja Zappe and Margit Cichna-Markl *

Table S1. Mean DNA methylation levels (%) of the *MGMT* promoter for all GBM cell lines obtained by PSQ and HRM by applying different calibration approaches. Calibration function: pol 3.

sample	mean methylation [%]			
	**PSQ (CpGs 72–83)**	**classical calibration (71.3–82.7 °C)**	**classical calibration (74.8–79.0 °C)**	**novel calibration (74.8–79.0 °C)**
GBM01	0.0	0.4	0.1	0.2
GBM10	0.0	0.3	−0.2	−0.2
GBM11	0.0	0.3	−0.3	−0.3
GBM12	0.0	0.0	−0.1	−0.2
GBM13	0.0	0.4	0.0	0.0
GBM14	0.0	0.5	0.0	0.0
GBM15	0.0	−0.1	−0.1	−0.2
GBM16	0.0	0.4	0.6	0.7
GBM17	0.0	0.1	−0.4	−0.4
GBM18	0.0	0.0	−0.5	−0.6
GBM19	0.0	−0.1	−0.4	−0.5
GBM20	0.0	−0.1	−0.5	−0.6
GBM21	0.0	0.0	−0.5	−0.6
GBM22	0.0	0.1	−0.4	−0.4
GBM24	0.0	0.2	0.0	0.0
GBM33	0.0	0.3	0.0	0.0
GBM36	0.0	0.8	0.5	0.5
GBM37	0.0	0.5	0.3	0.4
GBM38	0.0	0.4	−0.1	0.0
GBMm01	0.0	0.4	0.0	0.1
GBM05	2.7	2.5	5.7	5.4
GBM31	8.8	5.4	8.7	8.6
GBM29	11.8	11.3	14.6	15.3
GBM09	18.1	15.3	18.7	19.4
GBM32	22.5	13.3	16.3	21.8
GBM30	39.3	22.4	30.6	37.0
GBM23	50.7	30.5	44.3	52.6
T98G	60.8	61.4	61.4	61.3
GBM08	66.8	41.2	57.0	62.6
GBM03	72.7	50.4	75.1	78.2
GS01	73.5	50.8	68.9	72.0
GBM06	75.4	63.5	75.8	76.6
GBM25	78.4	57.1	76.7	78.3
GBM35	79.4	61.1	79.2	81.1
GBM28	80.7	60.8	80.5	82.2
GBM02	81.6	64.2	79.0	80.6
GBM34	82.7	64.9	79.8	80.8
GBM04	83.9	66.4	81.0	82.4

sample	mean methylation [%]			
	PSQ (CpGs 72–83)	classical calibration (71.3–82.7 °C)	classical calibration (74.8–79.0 °C)	novel calibration (74.8–79.0 °C)
GBM27	86.2	69.6	84.3	85.6
GBM07	91.2	79.7	87.7	88.2
GBM26	96.4	89.5	93.2	93.3

Table S2. Mean DNA methylation levels (%) of the *MGMT* enhancer 2 for all commercial cell lines obtained by PSQ and HRM by applying different calibration approaches. Calibration functions: pol 3 for PSQ and Hill for HRM.

sample	mean methylation [%]			
	PSQ (CpGs 11–18)	classical calibration (69.8–82.6 °C)	classical calibration (74.8–77.4 °C)	novel calibration (74.8–77.4 °C)
AU565	1.5	−2.7	0.7	0.6
T-47D	6.0	−5.4	0.2	−0.3
Hs 578T	16.6	−6.7	3.7	2.9
SK-BR-3	18.3	−3.2	6.3	6.3
ZR-75-1	31.0	12.0	27.3	27.9
HeLa	37.9	14.5	30.1	31.4
BT-549	47.8	52.7	66.3	65.2
CAMA-1	51.7	41.9	58.2	57.7
MDA-MB-468	52.3	59.9	70.8	69.0
CAL-51	60.9	40.0	56.1	56.4
MDA-MB-435S	68.7	44.8	68.4	68.0
KPL-1	76.0	68.4	77.0	75.2
MCF7	76.0	69.2	77.6	75.8
MDA-MB-231	80.4	68.8	77.0	75.3
BT-474	91.6	83.7	87.6	86.7
MCF 10F	92.0	88.9	90.7	90.1
MDA-MB-453	93.5	89.0	91.8	91.2
HCC1937	94.0	89.6	91.7	91.0
MCF 10A	94.7	87.2	92.2	91.7
HCC1143	95.6	95.2	95.5	95.2

Table S3. Mean DNA methylation levels (%) of the *MGMT* enhancer 2 for all GBM cell lines obtained by PSQ and HRM by applying different calibration approaches. Calibration functions: pol 3 for PSQ and Hill for HRM.

sample	mean methylation [%]			
	PSQ (CpGs 11–18)	classical calibration (69.8–82.6 °C)	classical calibration (74.8–77.4 °C)	novel calibration (74.8–77.4 °C)
GBM29	27.8	15.2	17.8	18.8
GBM02	53.9	73.5	80.6	77.2
GBM12	55.8	48.8	49.7	49.8
GBM13	58.2	66.9	76.3	73.9
GBM32	70.1	73.7	80.6	78.3

sample	mean methylation [%]			
	PSQ (CpGs 11–18)	classical calibration (69.8–82.6 °C)	classical calibration (74.8–77.4 °C)	novel calibration (74.8–77.4 °C)
GBM04	73.7	77.5	83.5	81.1
GBM27	74.8	70.2	77.6	76.0
GBM01	76.7	72.5	79.2	77.4
GBM17	80.4	88.3	92.3	91.2
GBM16	81.5	73.9	80.1	78.8
GBM21	84.8	74.4	80.6	79.5
GBM30	85.2	77.3	82.5	81.7
GBM15	85.4	86.2	89.8	88.5
GBM31	85.5	88.0	91.2	90.1
GBM26	86.0	89.8	94.1	93.3
GBM06	86.3	86.3	89.5	88.7
GBM18	87.0	77.0	83.0	82.1
GBM20	89.7	87.0	89.9	88.8
GBM28	89.8	86.4	89.9	88.7
GBM34	90.5	87.8	89.8	88.9
GBM07	90.7	87.4	90.3	89.4
GBM09	90.8	90.8	93.1	92.0
GBM22	90.9	92.9	95.1	94.5
GBM36	91.8	92.0	93.1	92.6
GBM11	92.1	91.9	93.7	93.1
GBM24	92.3	92.9	93.9	93.4
GBM08	92.8	91.5	93.1	92.6
GBM10	93.2	91.7	93.5	93.0
GBM14	93.4	92.5	93.6	93.0
GBM33	93.5	90.1	92.9	92.3
GBMm01	94.1	95.6	95.8	95.5
GBM19	94.2	94.6	95.5	95.2
GBM37	94.2	95.9	96.1	95.8
T98G	95.0	94.4	95.2	94.7
GBM35	95.5	94.0	95.5	95.1
GBM05	95.8	94.7	95.7	95.3
GS01	96.4	95.6	96.4	96.0
GBM03	96.7	96.8	97.3	97.1
GBM25	97.2	95.9	96.5	96.3
GBM38	97.3	97.9	97.8	97.5
GBM23	98.3	96.7	97.4	97.1

Table S4. Mean DNA methylation levels (%) of the *MGMT* enhancer 3 for all commercial cell lines obtained by PSQ and HRM by applying different calibration approaches. Calibration function: pol 3.

sample	mean methylation [%]			
	PSQ (CpGs 15–22)	classical calibration (71.5–80.1 °C)	classical calibration (75.7–76.5 °C)	novel calibration (75.7–76.5 °C)
AU565	−0.1	−0.6	−1.1	−1.1
SK-BR-3	−0.1	−1.7	−0.6	−0.7
CAL-51	1.9	−3.3	3.1	3.2
CAMA-1	1.9	−8.4	0.7	0.6
ZR-75-1	6.8	−5.1	9.4	9.5
BT-549	9.1	−18.1	6.5	6.5
MDA-MB-468	9.2	−10.1	9.5	9.6
T-47D	9.6	−6	10.9	11
MDA-MB-435S	12.6	−4.6	14.3	14.4
Hs 578T	14.6	−8.9	13.4	13.4
HeLa	23.1	−2.4	19.6	19.7
MDA-MB-231	29.8	2.4	25.3	25.4
MCF7	52.3	35.1	39.9	40.1
MCF 10F	60.6	41.2	44.9	45.1
BT-474	63.5	43	49.6	49.9
KPL-1	77.6	51.2	55.3	55.5
MDA-MB-453	81	56.2	61.6	61.8
MCF 10A	83.1	61.4	65.5	65.7
HCC1937	96.8	100.7	99.8	99.8
HCC1143	99.1	93.1	94.8	94.8

Table S5. Mean DNA methylation levels (%) of the *MGMT* enhancer 3 for all GBM cell lines obtained by PSQ and HRM by applying different calibration approaches. Calibration function: pol 3.

sample	mean methylation [%]			
	PSQ (CpGs 15–22)	classical calibration (71.5–80.1 °C)	classical calibration (75.7–76.5 °C)	novel calibration (75.7–76.5 °C)
GBM28	0.2	1.2	2.5	2.7
GBM30	2.5	0.4	3.3	2.9
GBM08	3.9	−2.1	−0.5	−0.9
GBM02	4.6	−0.3	2.4	1.8
GBM04	4.8	−1.1	1.8	1.2
GBM05	8.4	0.8	1.7	1.1
GBM17	8.4	3.0	11.6	11.9
GBM22	8.5	0.5	6.6	5.7
GBM18	9.1	1.9	7.9	7.8
GBM06	9.2	2.7	3.4	2.8
GBM31	10.3	7.1	15.4	16.0
GBM26	11.0	1.8	11.6	11.6

sample	mean methylation [%]			
	PSQ (CpGs 15–22)	classical calibration (71.5–80.1 °C)	classical calibration (75.7–76.5 °C)	novel calibration (75.7–76.5 °C)
GBM25	11.2	0.9	11.4	11.4
GBM01	11.3	−2.3	4.1	2.9
GBM32	11.3	4.9	12.8	13.1
GBM21	12.6	3.1	10.3	10.1
GBM33	12.9	3.2	11.4	11.3
GBM35	16.1	4.6	16.5	16.6
GBM16	16.5	1.9	11.6	10.2
GS01	19.0	0.7	12.5	10.4
GBM14	19.2	1.5	9.9	8.0
GBM13	22.9	9.2	18.7	18.4
GBM03	27.4	15.3	27.6	28.0
GBM19	30.2	9.1	18.2	16.6
GBM10	32.6	9.0	18.1	16.2
GBM27	34.5	15.5	28.2	28.4
GBM11	35.2	21.6	26.7	26.4
GBM34	36.1	20.7	28.7	28.7
GBM29	37.5	19.6	31.9	32.1
GBM07	42.2	13.4	24.1	22.9
GBM12	49.0	49.3	48.0	48.1
GBM23	73.9	51.5	61.5	61.6
GBM09	75.9	57.5	69.3	69.6
GBM20	75.9	57.5	69.2	69.7
GBM15	80.1	61.8	72.9	73.1
T98G	83.2	72.3	82.0	82.4
GBM24	85.6	86.5	89.8	89.8
GBMm01	86.7	67.8	80.7	81.0
GBM36	87.9	81.4	88.6	88.7
GBM37	91.2	92.3	92.8	92.7
GBM38	91.7	92.7	94.0	93.9

Table S6. Characteristics of the target regions.

region	primer sequence (5´→3´)	length [bp]/ CG content [1] [%]	Tm UM/ Tm M [°C]]	CpGs analyzed
promoter	F: GGATATGTTGGGATAGTT	98/47	75.1/80.5	72–83
98 CpGs	R: [Btn] CCCAAACACTCACCAAAT			
	S: GGATATGTTGGGATAGTT			
enhancer 2	F: [Btn] TTAAATAAGTGGTTTAGGTAGAGG	137/33	74.3/78.9	11–18
(Chen *et al*)	R: TACTAAACATTCCATTTCTAATTTCC			
46 CpGs	S: CCATTTCTAATTTCCTAACTC			
enhancer 3	F: TGTGTTAGTTTTAGTGGTTTAGA	138/38	75.5/78.1	15–22
(hs699)	R: [Btn] TAACACACAAACCAATCTCTC			
33 CpGs	S: TAGTTTTAGTGGTTTAGAAGT			

[Btn]: biotin, length: PCR product length; bp: base pairs, F: forward primer, PSQ: pyrosequencing, R: reverse primer, S: sequencing primer.
[1] of bisulfite converted DNA

Table S7. PCR conditions

**primer set**	**PCR mix**	**primer conc.**	**initial activation**	**PCR program**				**final elongation**
				**separation**	**annealing**	**elongation**	**cycles**	
promoter	mix 1	400 nM	95.0 °C, 5 min	94.0 °C, 30 sec	56.0 °C, 30 sec	72.0 °C, 30 sec	45	72.0 °C, 10 min
enhancer 2	mix 2	400 nM	95.0 °C, 15 min	94.0 °C, 10 sec	58.0 °C, 20 sec	68.0 °C, 20 sec	50	68.0 °C, 5 min
enhancer 3	mix 1	400 nM	95.0 °C, 5 min	94.0 °C, 30 sec	52.6 °C, 30 sec	72.0 °C, 30 sec	45	72.0 °C, 10 min

mix 1: 1x EpiTect HRM Master Mix (Qiagen) in RNase-free water
mix 2: 1x PCR master mix consisting of 2.5 U HotStarTaq DNA Polymerase (Qiagen) in 1x supplied PCR Buffer, 200 nM of each dNTP (PCR grade dNTP mix, Qiagen), and 1x EvaGreen dye (Biotium, USA) in RNase-free water
primer conc.: concentration of each primer
